# Oral quinolones versus intravenous β-lactam for the treatment of acute focal bacterial nephritis: a retrospective cohort study

**DOI:** 10.1007/s10096-024-04871-2

**Published:** 2024-06-10

**Authors:** L. Aceituno, A. Nuñez-Conde, J. Serra-Pladevall, B. Viñado, E. Castella, Laura Escolà-Vergé, C. Pigrau, V. Falcó, y O. Len

**Affiliations:** 1 Liver Unit, Internal Medicine Department, Vall d’Hebron University Hospital, Barcelona, Spain; 2 Internal Medicine Department, Mútua Terrassa University Hospital, Terrassa, Barcelona, Spain; 3 Microbiology Department, Vic Hospital, Vic, Spain; 4 Microbiology Department, Vall d’Hebron University Hospital, Barcelona, Spain; 5 Radiology Department, Vall d’Hebron University Hospital, Barcelona, Spain; 6 Infectious Diseases Unit, Medicine Department, Hospital de la Santa Creu i Sant Pau, Barcelona, Spain; 7 CIBERINFEC, Instituto de Salud Carlos III, Barcelona, Spain; 8 Infectious diseases Department, Vall d’Hebron University Hospital, Barcelona, Spain; 9 Medicine Department, Universitat Autònoma de Barcelona, Barcelona, Spain

**Keywords:** Urinary tract infections, Acute focal pyelonephritis, Β-lactam, Quinolones

## Abstract

**Background:**

Evidence regarding the best antibiotic regimen and the route of administration to treat acute focal bacterial nephritis (AFBN) is scarce. The aim of the present study was to compare the effectiveness of intravenous (IV) β-lactam antibiotics versus oral quinolones.

**Methods:**

This is a retrospective single centre study of patients diagnosed with AFBN between January 2017 and December 2018 in Hospital Universitari Vall d’Hebron, Barcelona (Spain). Patients were identified from the diagnostic codifications database. Patients treated with oral quinolones were compared with those treated with IV β-lactam antibiotics. Therapeutic failure was defined as death, relapse, or evolution to abscess within the first 30 days.

**Results:**

A total of 264 patients fulfilled the inclusion criteria. Of those, 103 patients (39%) received oral ciprofloxacin, and 70 (26.5%) IV β-lactam. The most common isolated microorganism was *Escherichia coli* (149, 73.8%) followed by *Klebsiella pneumoniae* (26, 12.9%). Mean duration of treatment was 21.3 days (SD 7.9). There were no statistical differences regarding therapeutic failure between oral quinolones and IV β-lactam treatment (6.6% vs. 8.7%, *p* = 0.6). Out of the 66 patients treated with intravenous antibiotics, 4 (6.1%) experienced an episode of phlebitis and 1 patient (1.5%) an episode of catheter-related bacteraemia.

**Conclusions:**

When susceptible, treatment of AFBN with oral quinolones is as effective as IV β-lactam treatment with fewer adverse events.

## Introduction

Acute focal bacterial nephritis (AFBN) was initially described by Rosenfield in 1979 [1]. It denotes a localized infection within the kidney parenchyma, characterized by the absence of tissue liquefaction. The majority of cases result from ascending urinary tract infections [1]. AFBN exhibits a higher prevalence among females, and predisposing factors include diabetes, immunosuppression, vesicoureteral reflux, and prolonged urinary tract catheterization [2]. Previous research has identified *Escherichia coli* as the most common pathogen causing AFBN, followed by *Klebsiella pneumoniae*, *Proteus mirabilis*, and *Pseudomonas aeruginosa* [3].

Currently, there is no definitive recommendation regarding the optimal antibiotic regimen or treatment duration for AFBN [4]. Historically, intravenous administration of third-generation cephalosporins has been the preferred antibiotic therapy [5]. Some authors suggest that intravenous antibiotics should be continued until resolution of fever and flank pain [6]. However, intravenous administration has inherent drawbacks, including the requirement for an intravenous catheter that carries an elevated risk of infection and thrombosis, among other complications [7]. Additionally, it leads to prolonged hospital stays or necessitates outpatient parenteral antimicrobial therapy (OPAT) programs, which impact both patient quality of life and healthcare system costs [8]. In this context, oral fluoroquinolones may offer a suitable alternative due to their high oral bioavailability and a large volume of distribution [9]. These agents have demonstrated efficacy in the treatment of acute pyelonephritis [10].

To date, no clinical trials have investigated the optimal therapeutic approach for patients with AFBN. Consequently, the objective of this study was to compare oral quinolones with intravenous β-lactam antibiotics in terms of clinical outcomes and rates of infection relapse, aiming to determine the most effective treatment option for AFBN.

## Materials and methods

### Study design, participants and setting

A retrospective cohort observational study was conducted at Vall d’Hebron University Hospital, a teaching hospital in Barcelona, Spain, with a 1,100 beds capacity. The study included adult patients (above 18 years of age) who were diagnosed with AFBN between January 2017 and December 2018. Patients were identified using the diagnostic codifications database. Only patients who met the diagnostic criteria for AFBN were included, and cases of infections occurring during pregnancy or puerperium, as well as renal abscesses, were excluded. Initial patient treatment followed the hospital protocol. Briefly, cefuroxime 500 mg/8 h IV or ceftriaxone 1 gr/day IV was administered for patients without suspected resistance, ertapenem 1 g/day IV for patients with suspected resistance but not severe infection and, finally, meropenem 1 gr/8 hours IV + amikacin 1 gr/day for patients with suspected resistance and severe infection. Upon availability of urine and blood culture results, patients were divided into two groups guided by antibiogram results: those who received intravenous β-lactam antibiotic, and those who received oral quinolone. The primary outcome was therapeutic failure, defined as the development of abscess, death, or relapse within 30 days of diagnosis. Adverse events were reported as secondary outcomes.

### Data collection

Demographic data, comorbidities, clinical information, and microbiological data were retrospectively collected from electronic medical records and entered in a database specifically created for this study.

### Definitions

AFBN was defined as the presence of at least one of the following symptoms: temperature above 37.8ºC, flank pain, or symptoms indicating lower urinary tract infection (dysuria, urgency, increased frequency, and pelvic pain) along with the identification of a rounded area with diminished or poorly enhanced echogenicity on kidney ultrasound or computed tomography scan [11].

Chronic kidney disease was defined as a glomerular filtration rate below 60 mL/min/1.73 m² calculated using the CKD-EPI (Chronic Kidney Disease Epidemiology Collaboration) score. Urinary tract abnormality included any functional or structural abnormalities, such as lithiasis, urinary incontinence, single kidney, urinary tract abnormalities, permanent urinary catheter, double J catheter, urostomy, benign prostatic hyperplasia, neurogenic bladder or cystocele. Recurrent urinary tract infections (UTIs) were considered when a patient was treated for UTI at least three times during the previous 12 months. Antibiotic treatment in the previous 3 months included any antibiotic received for any reason other than the current infection. A healthcare-associated infection was defined as an infection in patients who received wound care or skilled nursing care at home in the 30 days prior to the episode, those on hemodialysis, those hospitalized for two or more days at home in the 30 days prior to the episode, those institutionalized in a long-stay residence, or those who underwent an invasive urinary tract procedure in the 30 days prior to the episode or had a permanent urinary catheter [1].

Relapse was defined as the reappearance of symptoms and signs of urinary tract infection after completion of antibiotic treatment within the first 30 days.

### Microbiology and antimicrobial susceptibility data

A positive urine culture was defined as the isolation of an uropathogen at 104 CFU/ml or 103 CFU/ml if an indwelling catheter was present [3]. In cases where two microorganisms were isolated, both suggestive of uropathogens, and accompanied by pyuria and/or clinical symptoms, the urine culture was considered positive. Antimicrobial susceptibility was performed by microdilution (Vitek 2 Systems, bioMérieux, France). The minimum inhibitory concentration (MIC) values of the following antibiotics were interpreted according to the criteria established by the European Committee for Antimicrobial Susceptibility Testing (EUCAST) 2012 (version 2.0) guidelines (www.eucast.org): ampicillin, amoxicillin-clavulanic acid, piperacillin-tazobactam, cefuroxime, ceftazidime, cefotaxime, ertapenem, imipenem, amikacin, ciprofloxacin, trimethoprim-sulfamethoxazole, and fosfomycin. Isolates with intermediate category were considered resistant.

### Statistical analysis

Categorical variables were presented as percentages, while continuous variables were reported as means with standard deviations (SD) for normally distributed variables. The chi-square test or Fisher exact test, as appropriate, was used to compare categorical variables, and the student t-test was employed for continuous variables.

We analyzed the impact of the treatment strategy on the primary study outcome by two different approaches. First, predictors for the primary endpoint were assessed by logistic regression analysis that was performed to calculate crude and adjusted odds ratios (ORs) to identify risk factors for therapeutic failure. Variables that showed statistically significant differences in the univariate analysis were included in multivariate models. The models were constructed sequentially, starting with the variable most strongly associated with therapeutic failure and continuing until no other variable reached significance or changed the ORs of variables already in the model. The Hosmer and Lemeshow chi-square statistic test was used to assess the accuracy and goodness of fit of the prediction models. In a second analysis, and given the lack of randomization of treatment, a propensity score of receiving oral quinolones or IV β-lactam antibiotic was estimated using a backward stepwise logistic regression model that included all nonredundant variables with a P value ≤ 0.10 in the univariable analysis: gender, diabetes mellitus, chronic kidney disease, immunosuppression, kidney transplant, urological abnormalities, antibiotic treatment in the previous 3 months and infection associated with healthcare facilities. Propensity score matching was performed with a 1:1 ratio with replacement and a caliper of 0.05. Univariable logistic regression analysis was used to confirm the results of the primary outcome.

All statistical tests were two-tailed, and a P-value of 0.05 was considered statistically significant. Statistical analyses were conducted using IBM SPSS Statistics for Windows, Version 20.0 (IBM Corp, Armonk, NY).

### Ethics statement

The study was approved by the Ethics Committee of the Vall d’Hebron Research Institute under registration code PR (AG) 45/2019. The Ethics Committee reviewed our study and waived the need for informed consent, as all data and samples were analysed retrospectively and collected as part of routine clinical practice in accordance to current guidelines.

## Results

### Demographic and clinical data

**Fig. 1:**
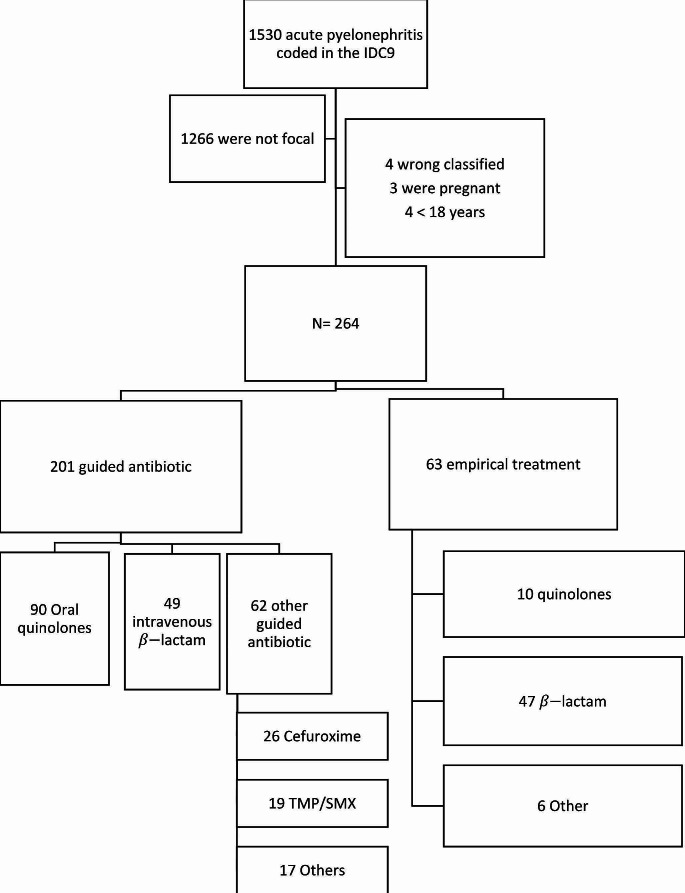
Flowchart of patient included in the study

During the period from January 2017 to December 2018, a total of 1,530 cases of acute pyelonephritis were diagnosed. Among them, 264 cases met the inclusion criteria (Fig. 1). Table 1 provides detailed information on demographic data and comorbidities. Of the included patients, 222 (84.1%) were women, and the mean age at diagnosis was 44.8 years with a standard deviation (SD) of 19.3 years. Among all patients, 41 (15.5%) had diabetes, 47 (17.8%) were renal transplant recipients, and 63 (23.9%) were receiving immunosuppressant treatment. Fifty (18.9%) patients had urological abnormalities, and 33 (12.5%) had recurrent UTIs. Table 1 highlights significant differences between the patients receiving oral quinolones and those receiving intravenous (IV) β-lactams. Patients treated with IV β-lactams had a higher frequency of diabetes mellitus, chronic kidney disease, kidney transplantation, immunosuppression other than transplantation, infections associated with healthcare facilities, and previous antibiotic treatment within the preceding 3 months.

**Table 1:** Epidemiological data, including patient characteristics and microorganisms found. General characteristics of patients treated with oral quinolones compared with those that received intravenous β-lactam (3rd generation cephalosporin, carbapenems)

	Patients N = 264	Oral quinolones N = 90	IV β-lactam antibiotics N = 49	p
**Demographics**				
Age in years, mean (SD)	44.8 (19.3)			
Woman	222 (84.1)	78 (86.7)	37 (75.5)	0.10
**Comorbidities**				
Diabetes mellitus 2	41 (15.5)	14 (14.6)	18 (36.7)	0.006
Chronic kidney disease	46 (17.4)	12 (13.3)	19 (38.8)	0.001
Immunosuppression	63 (23.9)	13 (14.4)	23 (46.9)	< 0.001
Kidney transplant	47 (17.8)	10 (11.1)	18 (36.7)	0.001
Urological abnormalities	50 (18.9)	10 (11.1)	14 (28.6)	0.017
Recurrent urinary tract infections	33 (12.5)	9 (10.0)	9 (18.4)	0.19
Antibiotic treatment in the previous 3 months	83 (31.1%)	14 (15.6)	24 (49.0)	< 0.001
Infection associated with healthcare facilities	32 (12.1)	5 (5.6)	11 (22.4)	0.005
***Aetiology*** (***n*** = ***201 positive cultures)***				
*Escherichia coli*	148 (73.6)	75 (83.3)	28 (57.1)	0.001
*Klebsiella pneumoniae*	26 (13)	5 (5.6)	14 (28.6)	< 0.001
*Proteus* spp	5 (2.5)			
Others (including *Pseudomonas aeruginosa*)	16 (7.9)			
Polymicrobial	6 (3)			
ESBL	15 (5.6)	3 (3.3)	12 (24.4)	< 0.001
Outcomes				
Therapeutic failure	17 (6.4)	6 (6.6)	5 (10.2)	0.9
Relapse	13 (4.9)	5 (5.5)	3 (6.1)	0.8
Deaths	2 (0.7)	0	2 (4.0)	
Evolution to an abscess	1 (0.37)	1 (1.1)	0	

Data are expressed as n (%). ESBL: extended-spectrum beta-lactamases

### Microbiological data

A urine culture was conducted for all 264 patients, and it yielded positive results in 199 cases (75.3%). The most isolated microorganism was *Escherichia coli*, identified in 148 cases (56.0%), followed by *Klebsiella pneumoniae* in 26 cases (9.8%) (Table 1).

Blood cultures were obtained from 234 patients, and 56 out of 234 (23.9%) yielded positive results. The etiology of AFBN was known for 201 episodes, with 199 cases attributed to positive urine cultures and 2 cases solely based on positive blood cultures.

Out of the 201 isolated microorganisms, 59 (29.3%) demonstrated resistance to ciprofloxacin, and 25 (12.3%) displayed resistance to third-generation cephalosporins. Among these resistant strains, 22 (88%) were also resistant to quinolones. Fifteen (7.4%) microorganisms produced extended-spectrum beta-lactamases (ESBL), while 9 (4.4%) produced AmpC enzymes. There was only one case (1.6%) of Klebsiella pneumoniae that produced OXA-48 type carbapenemase. Among the 148 *E. coli* isolates, 36 (24.3%) were resistant to quinolones, and 18 (12.1%) were resistant to third-generation cephalosporins. In contrast, *Klebsiella spp*. isolates exhibited resistance to quinolones in 46.1% (12 cases) and to third-generation cephalosporins in 76.9% (20 cases).

### Treatment

Initially, all patients received empirical intravenous treatment, with a mean duration of 5.7 days (SD 7.21). The most commonly administered antibiotics were cefuroxime (52.2%; 138/264), followed by carbapenems (17.8%; 47/264) and third-generation cephalosporins (9.8%; 26/264). However, empirical treatment was deemed inadequate in 30 patients (11.4%) (Table 2).

**Table 2:** List of empirical antibiotics and antibiogram-guided treatment administered in the cohort

	Total empirical treatment N = 264	Total antibiogram- guided N = 201	Oral antibiogram guided treatment N = 145	Intravenous antibiogram guided treatment N = 56
Cefuroxime	138	26 (12.9)	25 (17.2)	1 (3.8)
Carbapenems	47	30 (14.9)	0	30 (53.6)
Ceftriaxone	46	17 (8.5)	0	17 (30.4)
Other	16	6 (2.9)	5 (3.4)	1 (1.7)
Ciprofloxacine	10	94 (46.8)	88 (60.7)	6 (6.4)
Levofloxacin	0	2 (1.0)	2 (1.4)	0
Amoxicillin-clavulanate	7	6 (3.0)	6 (4.1)	0
TMP/SMX	0	19 (9.5)	19 (13.1)	0
Ceftazidim	0	1 (0.4)	0	1(1.7)

*Abbreviations* TMP/SMX trimethoprim-sulfamethoxazol

Data are expressed as n (%)

Once the antibiogram results were obtained for patients with known aetiology (201 cases), antibiotic therapy was adjusted accordingly. Among these patients, 49 (24.3%) received intravenous β-lactams and 90 (44.7%) were prescribed oral quinolones (Table 2). It is important to highlight that among patients treated with guided intravenous β-lactams, 33 (67.3%) were found to be non-susceptible to quinolones. The total mean duration of treatment was 21.3 days (SD 7.9). Specifically, the mean duration for quinolone treatment was 22.7 days (SD 9.0), while for IV β-lactams, it was 21.4 days (SD 8.1) (*p* = 0.47).

In terms of hospitalization days (including home medical care), the mean duration was of 5.4 days (SD 3.3) for the oral quinolones group, compared to 10.9 days (SD 8.1) for the IV β-lactams group (*p* < 0.001).

Among the patients affected by ESBL (15 cases), 12 were treated with intravenous carbapenems, while the remaining 3 received oral quinolones. For the 9 patients with AmpC-producing microorganisms, 8 were resistant to quinolone treatment and were prescribed carbapenems (3) or cotrimoxazole (2), among other alternative antibiotics.

### Therapeutic failure

In the group of patients who received guided antibiotic treatment, there were 11 cases of therapeutic failure out of 201 patients (5.4%). This included 8 cases of relapse (3.9%), 2 deaths within the first month (1%), and 1 case of progression to an abscess (0.4%). Specifically, among the patients treated with intravenous β-lactams (*n* = 49), 5 (10.2%) experienced therapeutic failure, while among those treated with oral quinolones (*n* = 90), 6 (6.6%) experienced therapeutic failure. However, there were no statistically significant differences in therapeutic failure rates between the two groups (*p* = 0.9). The consistency of this result was confirmed after propensity score matching. We observed no difference in the primary endpoint between the groups either when using weights inversely proportional to the probability of receiving oral quinolones treatment with the entire sample (oral quinolones vs. IV β-lactam antibiotic; OR 0.96, CI 95% 0.25 − 3.7, p 0.952) or paired cases (OR 0.33, CI 95% 0.03–3.2, p 0.341). However, it should be pointed out that there were only 8 paired cases after matching.

In the entire cohort, which included patients with and without microbiological identification and guided antimicrobial treatment, there were 17 cases of therapeutic failure out of 264 patients (6.4%). Four cases (6.4%) occurred in patients who received non-guided antibiotic treatment, while 13 cases (6.4%) occurred in the guided antibiotic treatment group. There were no statistically significant differences in therapeutic failure rates between these two groups (*p* = 0.9). Among the 62 patients in whom antibiotic treatment was not guided, those four who experienced therapeutic failure were administered oral cefuroxime (2 cases), oral ciprofloxacin (one case), and ertapenem (another case).

Among overall the cohort 17 patients experienced therapeutic failure at 30 days, 8 (47%) were immunocompromised, 7 (41.1%) had previous recurrent urinary tract infections, 10 (58.8%) had received antibiotic treatment in the previous months, and/or 5 (29.4%) were associated with healthcare facilities. Both patients who died presented a septic shock due to non-resistant *E. coli* and received intravenous carbapenem: one had a metastatic malignancy and the other had bilateral urostomies and a urothelial cancer in progression. The patient whose infection progressed to an abscess initially received intravenous cefuroxime. Due to persistence of fever, an ultrasound was performed on day 3 of treatment, which identified a 16 mm abscess. The antibiotic was then switched to intravenous ceftriaxone for 3 days, and after hospital discharge, the patient completed 4 weeks of treatment with oral quinolone, resulting in a favourable outcome. One patient in the non-guided antibiotic treatment group required a change in the antibiotic from oral ciprofloxacin to amoxicillin-clavulanate due to skin rash secondary to the former.

Regarding adverse effects related to antibiotic treatment (summarized in Table 3), they were significantly more frequent in the IV β-lactam treatment group (20.4% vs. 2.2%, *p* = 0.03). Among the patients who received IV β-lactam treatment: five (10.2%) experienced phlebitis, two (4%) had a mild allergic rash, two (4%) developed catheter-related bacteraemia and one (2%) had encephalopathy (related to ertapenem). In contrast, among the patients who received oral quinolones, one (1.1%) had a mild allergic rash, and one (1.1%) presented encephalopathy with ciprofloxacin.

**Table 3:** List of adverse events related to the guided treatment

	Total antibiogram- guided N = 201	Oral quinolones N = 90	IV β-lactam antibiotics N = 49	p
Treatment complications	12 (5.9)	2 (2.2)	10 (20.4)	0.03
Phlebitis	5 (2.5)	0	5 (10.2)	0.005
Skin allergy	3 (1.5)	1 (1.1)	2 (4.1)	0.69
Catheter-related bacteraemia	2 (0.9)	0	2 (4.1)	0.12
Encephalopathy	1 (0.5)	0	1 (2.0)	0.35
Hallucinosis	1 (0.5)	1 (1.1)	0	1
**Severity of adverse effect**				
	N = 12	**Oral quinolones** N = 90	**IV β-lactam antibiotics** N = 49	p
**Mild** reaction: symptomatic treatment	5 (41.6) (phlebitis)	0	5 (10.2)	0.005
**Moderate** reaction: lead to antibiotic switch	3 (25.0) (skin allergy)	1 (1.1)	2 (4.1)	0.38
**Severe** reaction: lead to admission and antibiotic switch	4 (33.4) (catheter-related bacteraemia, hallucinosis and encephalopathy)	1 (1.1)	3 (6.1)	0.24

Data are expressed as n (%)

We performed a univariable analysis (Table 4) to investigate the risk factors associated with therapeutic failure. Chronic renal disease (OR 2.82; CI 95% 0.98–8.07; *p* = 0.05), immunosuppression other than transplantation (OR 3.1; CI 95%1.14–8.42; *p* = 0.02), renal transplantation (OR 2.7; CI 95%0.95–7.82; *p* = 0.06), antibiotic treatment in the previous 3 months (OR 3.4; CI 95%1.24–9.29; *p* = 0.01) and infections associated with healthcare facilities (OR 3.38; CI 95%1.10-10.33; *p* = 0.03) were found to be related to therapeutic failure. However, in the multivariate analysis, the only risk factor associated with therapeutic failure was antibiotic treatment in the previous 3 months, although without statistical significance (Table 4).

**Table 4:** Univariable and multivariable analysis for therapeutic failure

	Univariable			Multivariable		
	OR	CI 95%	p	OR	CI 95%	p
Chronic renal disease	2.82	0.98–8.07	0.05			
Immunosuppression other that kidney transplantation	3.1	1.14–8.42	0.02			
Renal transplant recipient	2.7	0.95–7.82	0.06			
Antibiotic treatment in the previous 3 months	3.4	1.24–9.29	0.01	2.73	0.94–7.87	0.06
Infection associated with healthcare facilities	3.38	1.10-10.33	0.03			

## Discussion

The results of our study showed that both oral quinolone and IV β-lactam are effective treatments of AFBN when the causative microorganism is susceptible.

Our cohort’s baseline epidemiological data is similar to others AFPN series published [2, 12], affecting in > 75% of the cases women with a median age of 45 years and similar proportion of patients with urological abnormalities, but we included more immunosuppressed patients and bacteraemia was more frequent. The microbiology in our cohort resembles that described previously [2, 12], with Escherichia coli being the most frequent aetiology, and with a similar percentage of ESBL-producer’s microorganisms in the whole cohort around 5% [12]. In our cohort, the resistance rates for fluoroquinolones and third-generation cephalosporins in E. coli were 24% and 12%, respectively. Another Spanish study that analysed the antimicrobial susceptibility of Gram-negative organisms involved in urinary tract infections found a 14% of ESBL producers [13], and a similar large multicentre study performed in in the United States evaluating antibiotic resistance rates among urinary tract isolates of E. coli found resistance to ciprofloxacin in 25.8% of the isolates, and 15.7% were ESBL producers [14], with co-resistance rates between fluoroquinolones and third generation cephalosporins higher than 80%, similar to ours.

Current guidelines acknowledge the lack of high-quality studies for patients with AFBN and rely on expert advice to recommend treating with the same antibiotics as for uncomplicated acute pyelonephritis but for a longer duration [6]. Ciprofloxacin has previously shown its efficacy in treating bacterial acute pyelonephritis. In fact, Sandbert T et al. [10] demonstrated that treatment can be reduced from 14 to 7 days. It has also shown its utility in treating *Klebsiella pneumoniae* liver abscesses in a stepdown oral fashion compared to IV ceftriaxone for the whole course of the treatment [10]. Similar results have been shown by Navasa et al. [15] in the treatment of spontaneous bacterial peritonitis, and same results were shown in the study performed by Tuncer et al. in 2006 [16, 17].

Both treatment alternatives were effective in treating AFBN, with a high rate of recovery in both treatment groups and few cases of relapse, similar to other cohorts of AFBN [2, 12, 18] or complicated urinary tract infections [19]. However, it is worth noting that patients treated with intravenous β-lactams had significantly more comorbidities, previous antibiotic use, and a higher prevalence of ESBL. Therefore, a propensity score matching analysis was performed, yielding the same results, albeit with only 8 matched cases. As the decision to treat the patients depended on the physician at charge, it seemed as if they were more confident with IV β-lactam with this kind of patients, even if the isolated microorganism was susceptible to quinolones. Nonetheless, when we analyzed the risk factors associated with therapeutic failure, only the administration of antibiotics in the previous three months approached significance. The reasons behind this finding may be multiple. This variable probably reflects more complex patients, including those with immunosuppression or urologic abnormalities, who likely have more infections, with more resistant microorganisms, and therefore received more antibiotic treatment. This has been not studied in previous AFBN series, but in observational studies evaluating risk factors for mortality in urinary tract infections, the severity of illness and comorbidities seemed more important risk factors for mortality than the time to administration of antibiotics or inappropriate empirical antibiotic therapy [20].

Considering that both alternatives are equally effective, oral antibiotics offer the advantage of early discharge, which may be more convenient and cost-effective for patients. In fact, compared to the series of Jiao et al. in which most of the patients received an intravenous β-lactam for AFBN, mean hospital stay was shorter in the group of our cohort who received oral fluoroquinolones (19 days vs. 5.4 days). In addition, intravenous antibiotics have a significantly higher risk of complications compared to oral fluoroquinolones, such as phlebitis or bacteraemia, and are associated with a longer hospitalization [2]. Even in the case of OPAT, which also facilitates early discharge, some series report that 20% of patients experience vascular access device complications [21]. In fact, the benefit of intravenous antibiotics for many indications is theoretical and lacks robust evidence, as shown by Li HK et al. [22]. A recent multicentre observational cohort study comparing intravenous and oral antibiotic stepdown strategies for Gram-Negative bacteraemia in complicated urinary tract infections found no differences in effectiveness between fluoroquinolones, trimethoprim-sulfamethoxazole and intravenous β-lactams [23] A health economic study is needed to highlight even more the advantages of oral fluoroquinolones, which in our study seem to be safer and permit an early return home.

We performed a logistic regression to assess the impact of the confounding factors, highlighting the significant impact of recent antibiotic treatment on the outcome, underscoring the necessity of considering such treatments when evaluating patient prognosis and management strategies. Furthermore, in the context of other potential confounding variables, the exclusion of certain factors from the logistic regression model, suggests their lack of a statistically significant association with the outcome of interest.

Our study had several limitations. First, this is a single-hospital-based study. Second, due to the retrospective nature of the study and the use of codification database to identify patients, some information could have been not collected and some cases could have been dismissed because of incorrect codification. Third, there could have been some interobserver bias in the diagnosis of AFPN with abdominal ultrasound as not all the patients were diagnosed with CT. Finally, there were significant differences between groups as patients treated with IV β-lactam had more comorbid conditions.

Despite these limitations, the major strength of our study relies on the large cohort of adult patients with AFBN that represents real-world clinical practice including a high ratio of immunosuppressed patients and patients with all kinds of comorbidities.

In summary, the results of the present study indicate that, when the microorganism is susceptible, oral ciprofloxacin is a safe and effective option to treat acute focal bacterial nephritis with significant advantages over IV β-lactam such as convenience and cost savings. Further research is needed to validate these findings and to identify specific patient populations that may benefit from this treatment approach.

## Abbreviations

AFBN: Acute focal bacterial nephritis
CKD-EPI: Chronic Kidney Disease Epidemiology Collaboration
EUCAST: European Committee for Antimicrobial Susceptibility Testing
ESBL: Extended-spectrum beta-lactamases
OPAT: Outpatient parenteral antimicrobial therapy
ORs: Odds ratios
SD: Standard deviations
UTIs: Urinary tract infections
